# Nanofluids for Performance Improvement of Heavy Machinery Journal Bearings: A Simulation Study

**DOI:** 10.3390/nano10112120

**Published:** 2020-10-25

**Authors:** Hamid Sadabadi, Amir Sanati Nezhad

**Affiliations:** 1 College of Engineering and Technology, American University of the Middle East, Kuwait; 2 Center for Bioengineering Research and Education, BioMEMS and Bioinspired Microfluidic Laboratory, Department of Mechanical and Manufacturing Engineering, University of Calgary, Calgary, AB T2N1N4, Canada

**Keywords:** nanofluid lubricant, IF-WS_2_ nanoparticles, hydrodynamic journal bearing, CFD simulation, nanoparticle aggregation

## Abstract

Nanofluids have extensive applications in hydrodynamic journal bearings used in heavy industry machinery. Inorganic fullerene-like tungsten disulfide nanoparticles (IF-WS_2_ NPs) are the most common additive for lubrication purpose due to their excellent mechanical characteristics along with their effect on reducing friction and wear. In this work, a computational simulation approach with discrete phase modeling (DPM) of suspended nanoparticles was used to evaluate the application of the IF-WS_2_ nanofluid lubricant on load carrying capacity of high-load journal bearings where the normal loads are high, considering the bearing dimensions. For accurate simulation, nanofluid viscosity was calculated considering the aggregation effect of NPs by using scanning electron microscopy (SEM) imaging of the nanofluids. A benchmark study was first performed to assess the model accuracy. Hydrodynamic lubrication was simulated under different nanofluid weigh fractions. The simulated pressure distribution was then employed to determine the load capacity of the bearing. The results show an approximately 20% improvement of load carrying capacity at 5% weight fraction of WS_2_-oil nanofluid.

## 1. Introduction

Hydrodynamic journal bearings have been extensively used in heavy industry machines and turbomachinery [1] to support rotating shafts due to their superior durability, low maintenance cost, and excellent load carrying capacity [2,3,4,5,6]. Journal Bearings are critical components of the tools operating downhole, such as directional drilling (DD) tools that are used in oil and gas exploration and drilling applications. During the drilling process of a bent hole, the entire DD tool is bent in the wellbore while the rotor rotates in its own place. In such common operational conditions, the normal force applied to each supporting journal bearing could easily exceed 10,000 N, even though their OD footprint is just a few centimeters. Therefore, finding new technologies to improve the load carrying capacity of these bearings to lower wear and subsequently to control geometrical tolerances is a critical task towards improved tool reliability [7].

Computational fluid dynamics (CFD) are generally used in the industry as a modeling technique to simulate and evaluate the performance of components with fluid mechanics involved, such as hydrodynamic bearings without a need for excessive prototyping and testing [5,8,9,10,11,12,13,14,15,16,17,18]. Manshoor et al. [10] used a 3D CFD simulation model to investigate the performance of thin-layer lubrication in journal bearings. They compared the effect of three different turbulence models on design parameters of bearings with various slenderness (L/D) ratios. Concli [15] developed a model for the numerical simulation of small journal bearings. The model was first validated with experimental data and then was used to investigate cavitation, bearing 3D effect and other performance metrics by employing an open-source CFD code. Using the same open-source code, Concli [16] proposed a state-of-the-art meshing approach for journal bearings that facilitate gridding of complex geometries with reduced number of meshes, while it improves the solution convergence.

An efficient way to reduce the wear of contact parts would be to improve the oil lubrication performance by surface modification and texturing [19,20] or by using nanofluids [2,3,4,21]–colloidal suspensions of nanoparticles (NPs) in the base oil. Over the past three decades, nanoparticles have been extensively studied as an additive for lubricants and have been used to reduce the friction coefficient between moving parts and consequently lower the wear [22]. Inorganic NPs are well suited for lubrication applications due to their superior compressive strength, whereas their shear strengths are minimal [14]. Tungsten disulfide nanoparticles (WS_2_ NPs) are undoubtedly the focal point of research around additive NPs for lubrication purposes because of their excellent tribological and mechanical properties [2,3,13,14,21,22,23,24,25]. Over the last two decades, WS_2_ NPs have spurred considerable interest in automotive industry applications in their nanotube form [3], fullerene-like form [14,24] or nanosheet form [25]. Their effect is mainly noticeable in mixed-film lubrication and boundary lubrication regimes, where mild to severe wear and damage can occur [13,14,26].

While the majority of researches evaluate the performance of nanoparticle additives in terms of thermal conductivity [4,27], friction reduction [4,13,26,28], and wear phenomena [29], there are very few that address the effect of nanofluids on load carrying capacity [24,25,30]. These works mainly use a standard reciprocating ring–block tester [24] or ball machine [25] as their test setup. Understanding the effect of nanofluids on load carrying capacity of bearings is important for design engineers to properly select a nanofluid lubricant based on the available geometry and the magnitude of the applied normal load. In this work, which is the first phase of an industrial research collaboration, we have investigated the effects of adding IF-WS_2_ NPs on the load carrying performance of journal bearings through a computational approach. To accurately execute the model, the viscosity of the nanofluid was calculated considering the aggregation of WS_2_ NPs in the medium. Then, the simulation was performed to obtain the pressure profile of hydrodynamic lubrication under various NP weight fraction percentages (wt%). Finally, by integrating the pressure profile, the load capacity of the bearing was obtained for each case.

## 2. Materials and Methods

### 2.1. Materials

A 1:1 ratio mixture of synthetic oil SynFilm GT320 and GT220 from Royal Purple Synthetic Oil (Porter, TX, USA), which is generally used in drilling tools, was used in this work. The steady-state temperature of the oil in the drilling tools under continuous operation downhole was around 100 °C. All the parameters and calculations were obtained assuming this constant temperature. For the nanoparticles, inorganic fullerene-like tungsten disulfide nanoparticles (IF-WS_2_ NPs) were purchased from US Research Nanomaterials Inc. (Houston, TX, USA). The NPs were spherical fullerenes with symmetrical and uniform structures, consisting of concentric curled layers between 20–100 layers. Their average diameter was 50 nm with diameter ranging between 30 nm and 70 nm. The density of the NPs was ρ = 7.5 gr/cm^3^.

### 2.2. Bearing Theory

**Figure 1:**
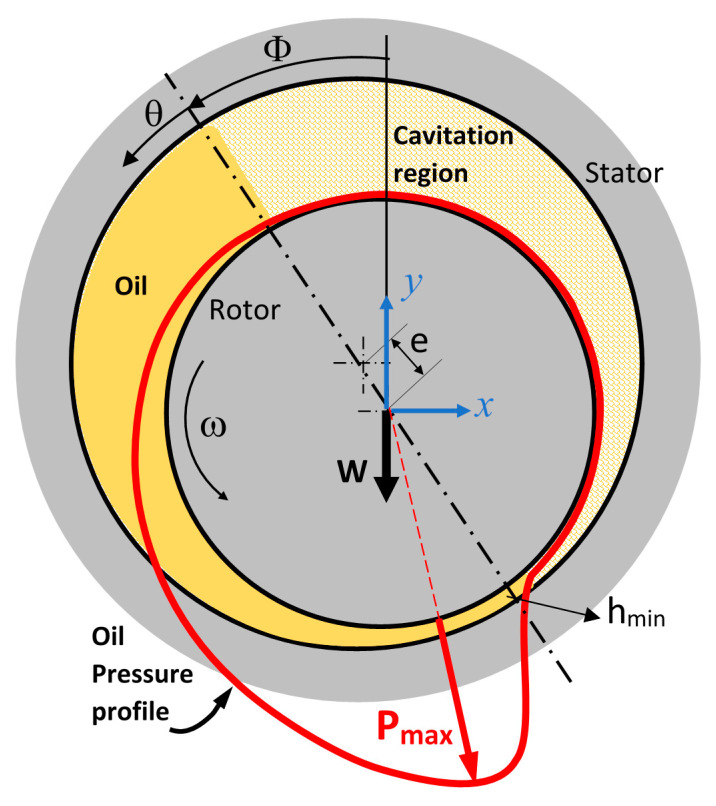
Basic journal bearing geometry and schematic stator pressure distribution profile.

For a journal bearing with the parameters given in schematic Figure 1, assuming negligible variation in viscosity at the constant temperature 100 °C, for steadily loaded condition where $\dot{\epsilon }=0$ and $\dot{\emptyset }=0$, the reduced form of Reynolds equation, Equation (1), as follows, can be used to evaluate the bearing performance [31,32,33]:

(1) $$\frac{\partial }{\partial \theta }\left[{\left(1+\epsilon \;\cos\;\theta \right)}^{3}\frac{\partial P}{\partial \theta }\right]+R^{2}\frac{\partial }{\partial z}\left[{\left(1+\epsilon \;\cos\;\theta \right)}^{3}\frac{\partial P}{\partial z}\right]=-6\eta \omega {\left(\frac{R}{C}\right)}^{2}\epsilon \;\sin\;\theta$$

where η is the coefficient of the lubricant viscosity in Pa s, U is journal surface velocity, *z* is the coordinate in axial direction, and ϵ=e/C is the eccentricity ratio. The closed form solution to Equation (1), is available as a function of slenderness ratio = L ⁄ D. For short bearings with low L/D ratio, the solution known as Ocvirk’s solution, Equation (2), and for long bearings with high L/D ratio, the closed form solution known as the Sommerfeld solution, Equation (3) [31]:

(2) $$P_{s}=P_{\mathrm{short}}\;\left(z,\theta \right)=\frac{3{\eta \mathrm{UL}}^{2}}{{\mathrm{RC}}^{2}}\left[\frac{1}{4}-{\left(\frac{z}{L}\right)}^{2}\right]\frac{\epsilon \;\sin\;\theta }{{\left(1+\epsilon \cos\;\theta \right)}^{3}}$$

(3) $$P_{L}=P_{\mathrm{long}}\;\left(\theta \right)=\frac{6\eta \mathrm{UR}}{C^{2}\left(2+\epsilon^{2}\right)}\left[\frac{\epsilon \;\sin\;\theta \;(2+\epsilon \;\cos\;\theta }{{\left(1+\epsilon \cos\;\theta \right)}^{2}}\right]$$

The analytical solution P_L_ is valid when the slenderness ratio = L/D is bigger than 2.0, while the solution P_s_ is applicable for a L/D ratios smaller than 0.25 [26,31,34]. For the cases where the L/D ratio range is within 0.25 to 2.0, both approximations are inaccurate. For such conditions, the empirical equation for film pressure, P(θ), proposed by Reason and Narang (P_RN_), is by far one of the best closed form solutions compared to other readily available solutions in the literature. This approximation uses a harmonic mean of the short bearing (P_s_) and long bearing (P_L_) solutions, given by Equation (4) [35]:

(4) $$\frac{1}{P_{\mathrm{RN}}\left(\theta ,z\right)}=\;\frac{1}{P_{L}\;\left(\theta \right)}+\frac{1}{P_{s}\;\left(z,\theta \right)}$$

This equation is an accurate approximation of film pressure, even compared to the numerical solutions for short L/D ratios. However, for higher L/D ratios, it overpredicts the pressure. For these cases, there are a few models that correct Equation (4) based on the slenderness ratio. One of the best is the empirical pressure weighting correction factor proposed by Hirani et al. [31] and Equation (4) is modified as $1/P_{RN}=g_{1}/P_{L}+g_{2}/P_{L}$ where weights $g_{1}=e^{{\left(1-\epsilon \right)}^{3\;}}\;and\;g_{2}=1+\epsilon {\left(L/D\right)}^{1.2\;}\left[e^{\epsilon^{5\;}}-1\right]$.

### 2.3. CFD Simulation of Hydrodynamic Lubrication

In drilling tool applications, the slenderness ratio L/D ≅ 2, so one can safely use the long bearing model for all the analyses (Sommerfeld solution, Equation (3)). This model also simplifies the simulation process as it enables 2D modeling and reduces costly CPU-time needed for a 3D simulation.

Computational fluid dynamics (CFD) were used to model the incompressible lubrication between the rotor and the stator. The full Navier–Stokes equations were solved with the commercially available software package, ANSYS Fluent (ANSYS, Canonsburg, PA, USA). The CFD model used in this work is based upon an actual journal bearing with parameters that are given in Table 1. A 2D geometry is used to model the bearing. For each simulation case (given in Table 2), the rotor axis was set in an eccentricity from stator axis based on its Sommerfeld number [36]. The problem is being treated as known geometry while we solve the model for the pressure value in the gridded (meshed) area.

For modeling of the flow regime, the realizable k−ε turbulence model proposed by Shih et al. [37] was utilized. This model improves the performance of the standard k−ε model by accurate estimation of the spreading rate of both planar and round jets. The realizable k−ε model has previously shown its merit for CFD simulations of journal bearings compared to other turbulence models [10]. The model constants $\sigma_{\varepsilon }$ and $C_{\varepsilon 2}$ have values of 1.2 and 1.9, respectively. Since NPs are much heavier than their surrounding lubricant, the effect of gravity on particles was also considered in the simulation. For the boundary condition, the rotor was considered as a moving wall with the angular velocity N along its axis.

For the meshing purpose, as the clearance size of the bearing in fluid domain is very small compared to rotor/stator radius, quad meshing method was preferred and used in the simulation. Generally, the result’s precision is linked to the refinement of the physical domain’s grid size. Prior to running the simulations, for one of the simulation cases (Case 1 with base lubricant without additive), a mesh size sensitivity examination was performed to ensure the results are independent of the mesh size.

For the modeling of WS_2_ NPs in the oil, discrete phase modeling (DPM) of nanoparticles was used in a fully-coupled condition to ensure the interaction between the flow and particles were considered. DPM consists of spherical particles dispersed in the continuous phase. The size of NPs used for DPM analysis was selected as the average size of aggregated WS_2_ NPs and obtained from scanning electron microscopy (SEM) images. Since these NPs were big and heavy compared to the sounding medium, the chance of particle collision is high; therefore, the particle interaction has been assumed for modeling. For the simulation purpose, five different weight fraction percentages (wf.%) of WS_2_ nanofluids were used, as shown in Table 2. Once the simulation was completed for each case, the distribution of static pressure on the nodes along the circumference of the rotor was obtained. For better visualization, the pressure graphs also drawn in polar format as schematically shown in Figure 1. The applied normal load induces a pressure distribution profile on the rotor. Inversely, the applied normal force can be obtained by the integration of the pressure distribution [15]. In the last step, to calculate the load carrying capacity, the directional polar integration (in y-direction) of the pressure profile was obtained by using a Riemann sum rectangle approximation method where in polar form the rectangles are replaced by sectors of a circle using Equation (5), as below:

(5) $$W_{y}=\;\int_{0}^{\pi }\left(\mathrm{PR}\;d\theta \right)\;\sin\;\theta =\overset{\theta =\pi }{\underset{\theta =0}{\sum }}P.R.\Delta \theta .sin\left(\theta \right)$$

**Figure 2:**
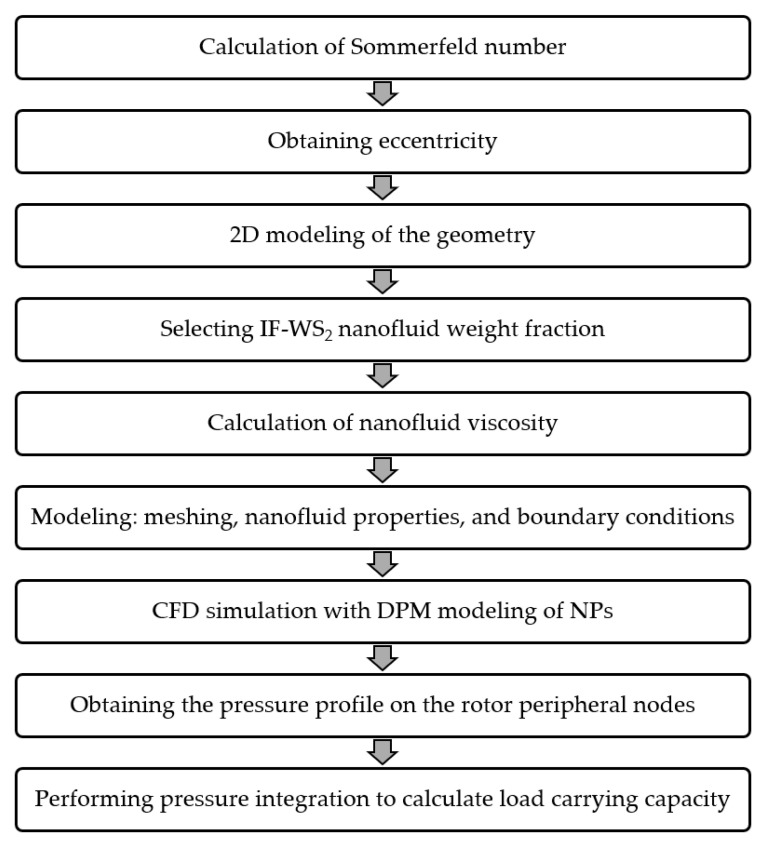
Process flowchart for CFD analysis of hydrodynamic bearing with nanofluid lubricant.

Figure 2 shows the process flowchart that was used in this work to conduct CFD modeling of hydrodynamic lubrication to obtain the pressure profile and finally to calculate the load capacity of journal bearings for each simulation case. 

### 2.4. Aggregation of WS_2_ NPs and Nanofluid Viscosity

Rheological properties of nanofluids are considerably affected by the aggregation of nanoparticles [4,13,27]. There are models that correlate relative viscosity $\eta_{r}$ with volume fraction ∅ of nanofluids [27,30]. Under a shear flow, when the applied hydrodynamic forces to an aggregate is sufficient, the force can break it down into smaller aggregates or individual nanoparticles. For the cases where these forces are small, aggregates become stable and form spherical shape units with active volume fraction ∅_a_ which is different from the nominal volume fraction. The modified Krieger–Dougherty equation, Equation (6), can be used to obtain the relative viscosity of nanofluids when stable aggregation is likely to form [27,38]:

(6) $$\frac{\eta_{nf}}{\eta_{bf}}={\left(1-\frac{\emptyset_{a}}{\emptyset_{m}}\right)}^{-\left[\eta \right]\emptyset_{m}}$$

where $\eta_{nf}$ is nanofluid viscosity, $\eta_{bf}$ is base fluid viscosity, [η] is intrinsic viscosity with the value of 2.5, $\emptyset_{a}$ is the effective or active volume fraction, and $\emptyset_{m}$ is the maximum volume fraction or maximum particle packing fraction of nanoparticles at which the flow can happen. $\emptyset_{m}$ varies from 0.5 for low shear rate and 0.605 for high shear rates. Chen et al. [28] proposed an accurate empirical equation for the calculation of $\emptyset_{a}$ as a function of NP aggregate size ratio, $\emptyset_{a}=\emptyset \;{\left(a_{a}⁄a\right)}^{\left(3-D\right)}$, where $a_{a}$ is the average size of aggregates and *a* is the size of individual nanoparticles. The parameter D, known as fractal index, is the measure of the extent of changes in the packing fraction from the center to the edge of the aggregates [4,39]. D has a typical value between 1.8 and 2.5 for aggregation with limited diffusion and between 2.0 and 2.2 for aggregation with limited reaction. For the case of IF-WS_2_, NPs tend to be in a stable and strong aggregated state in the nanofluid. For these cases, the parameter D is reported between 1.6 and 1.8 for nanofluids with spherical nanoparticles [4]. In the present work, the average value of D=1.7 is assumed for the analysis and therefore the Kriegere–Dougherty equation is reduced to Equation (7):

(7) $$\frac{\eta_{nf}}{\eta_{bf}}={\left(1-\frac{\emptyset }{0.605}{\left(\frac{a_{a}}{a}\right)}^{1.3}\right)}^{-1.25}$$

Therefore, the problem of finding the nanofluid relative viscosity reduces to finding the size of NP aggregates. To calculate the aggregate particle sizes, scanning electron microscope (SEM) imaging of nanofluid samples was used. The suspensions of synthetic oil and IF-WS_2_ NPs at 3.7 wt.% were thoroughly mixed using an ultrasound bath for 12 h, then 5 samples were collected and placed onto clean silicon wafers (University Wafer Inc., MA, USA). All NP samples on the surface of silicon wafers were examined by means of a commercial SEM (FEI XL30-30 kV SEM, Philips, Eindhoven, Germany). The images were used for statistical and image analysis and to detect possible variations. The most generic image is shown in Section 3.2. An in-house image processing program was then used to extract the size distribution graph of NPs from SEM images to obtain the curve-fitted size normal distribution [40,41,42].

## 3. Results and Discussions

### 3.1. Case-Study: CFD Model Evaluation

**Figure 3:**
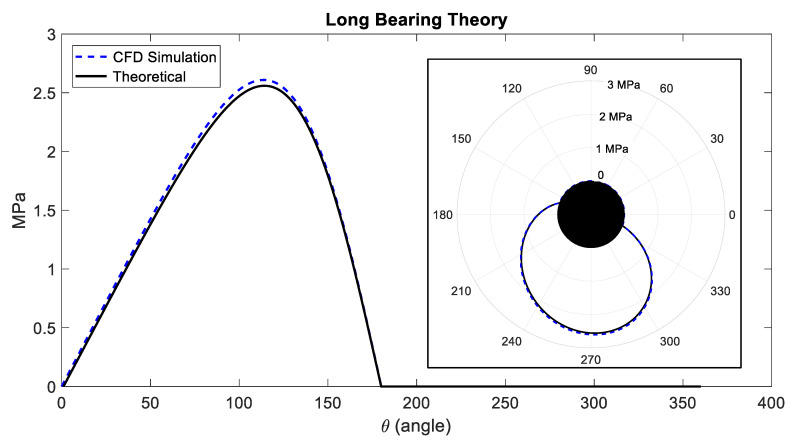
Comparison of the pressure profile from the theoretical model Equation (3) and CFD computational simulation.

A benchmark study is performed on the developed CFD model to evaluate the model and to assess the accuracy of the simulation results. For this purpose, pressure simulation results for Case 1 (with no NPs) were obtained and compared to the results from long bearing estimation of the Sommerfeld solution in Equation (3), as shown in Figure 3. The pressure distribution along the rotor circumference is shown in both regular graph and in polar form for easier interpretation. The results show that the proposed CFD model simulates the hydrodynamic lubrication process comparable to the direct solution. Compared with long bearing Sommerfeld solution, the CFD model overestimates the pressure with maximum 2.0% difference, which is considered to be acceptable for this analysis.

### 3.2. Characterization of NP Aggregation and Viscosity Calculation of the Nanofluid

**Figure 4:**
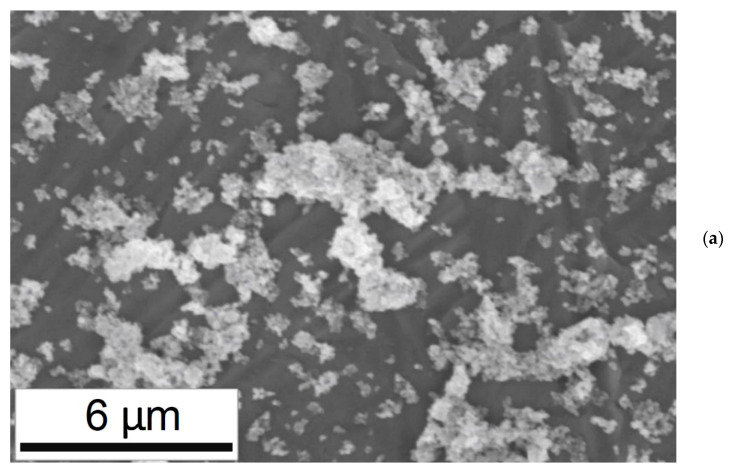

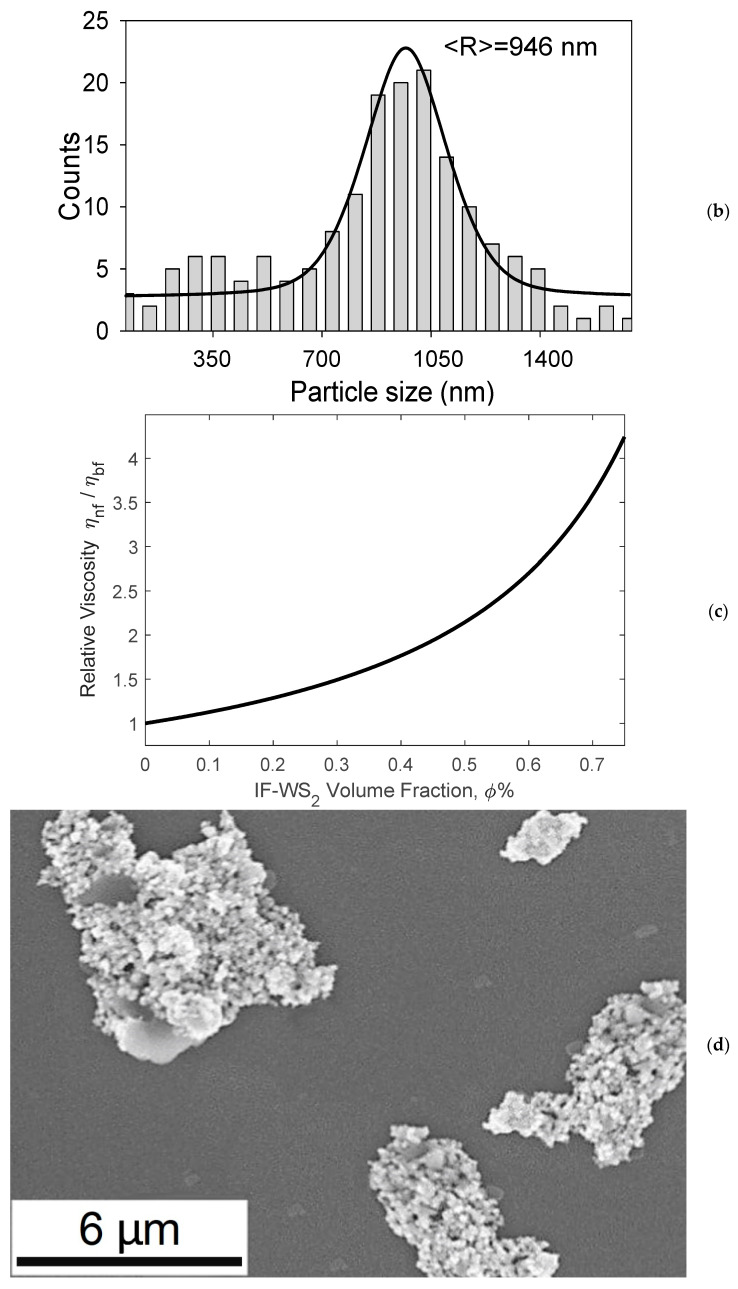
Characterization of the nanoparticle aggregation for evaluation of the viscosity of the nanofluid: (**a**) scanning electron microscopy (SEM) micrograph of the IF-WS_2_ NPs at 3.7 wt% on the surface of a silicon wafer; (**b**) Size distribution graph of NP agglomerates for 3.7 wt%. NP agglomerates have an average size of $a_{a}=946\pm 175\;\mathrm{nm}$; (**c**) Relative viscosity of the nanofluid as a function of NP Volume Fraction; (**d**) Extensive agglomeration of NPs is observed at high weight fractions. The image corresponds to 7.4 wt%.

To characterize the aggregation of IF-WS_2_ NPs and to obtain the average diameters of agglomerates, SEM images of the nanofluid samples with 3.7 wt% of NPs were obtained. Figure 4a shows a SEM image of the particles on the surface. The image shows that IF- WS_2_ particles are majorly agglomerated to a great extent while they also exist in a much lower quantity as individual NPs. Figure 4b shows the size distribution curve of the aggregates. The graph was obtained by the analysis of ten SEM images of the samples. The distribution graph shows that the agglomerates have an average size of $a_{a}=946\;\mathrm{nm}$ which is further used to predict nanofluid viscosity. This value of $a_{a}$ was also used in the DPM modeling of NPs in the simulation.

The relative viscosity of nanofluid as a function of volume fraction ∅ was calculated by using Equation (7) as shown in Figure 4c. Nanofluid viscosity is greatly affected by the parameter $a_{a}$, the average size of aggregates. Our investigations show that high aggregated NPs can be observed at high weight fractions. Specifically, for weight fractions below 4.9 wt%, the SEM images showing small variations in terms of particle aggregation. However for 6 wt% and above, the aggregations are bigger in size and it is unlikely to locate individual NPs or even small aggregates on SEM images. Figure 4d is showing SEM image of nanofluid sample at 7.4 wt%. This image shows NPs form very large agglomerates with size of few micrometers.

In agreement with our findings, previously published reports show that IF-WS_2_ NPs create strong agglomerations that make them very stable during hydrodynamic lubrication [13,22]. Such a property reduces the chance of having them break down during hydrodynamic lubrication. Therefore, weight fractions above 6.0 wt% deem unfeasible for practical lubrication purposes. 

### 3.3. Simulation Results

**Figure 5:**
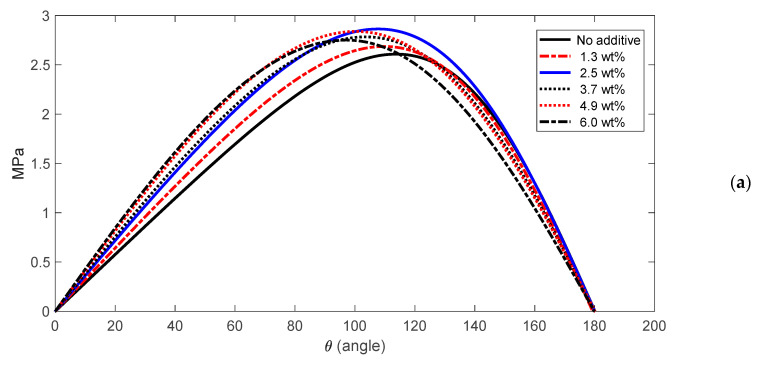

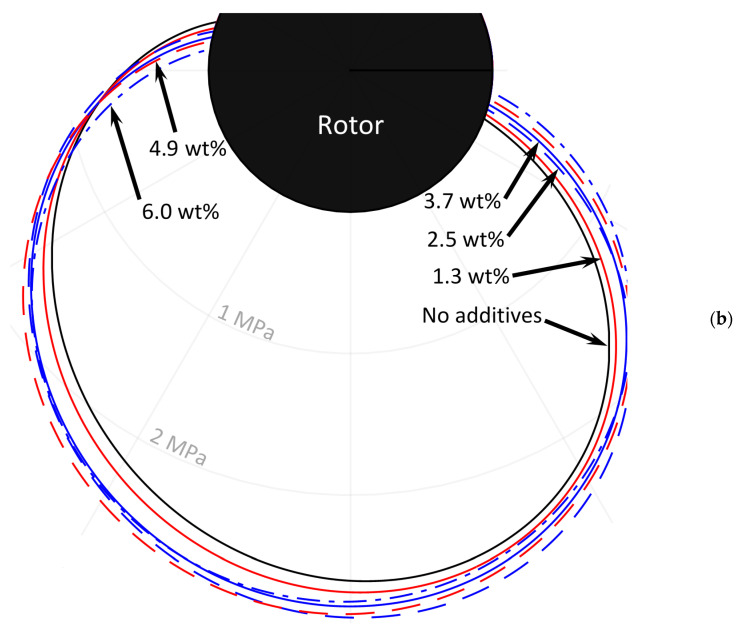
The pressure distribution profile of the journal bearings subject to different concentrations of the nanofluid: (**a**) Cartesian plot of P(θ); (**b**) Polar plot of P(θ).

The CFD model explained in Section 2.3 was used to simulate the effect of IF-WS_2_ nanofluids with different weight fractions (Table 2). The viscosity of the nanofluid, calculated based on Figure 4c, were used for hydrodynamic bearing simulations at different nanofluid concentrations. Then, the pressure profile was obtained for each case. The results of the pressure profile distribution along the rotor circumference are shown in Figure 5a for all six cases in Table 2. To simplify the result interpretation, Figure 5b shows the polar plot of the same data. The pressure data show that increasing the NP wt% normally increases both maximum pressure (*P_max_*) and shape of the pressure profile. The pressure profile become wider for higher wt%. However, there are cases where *P_max_* does not follow the growing trend. In general, higher maximum pressure and wider pressure profile leads to improved load capacity of the bearing. A wider pressure distribution results in a more uniform load distribution on the peripheral of the rotor and therefore it reduces localized wear/erosion of the rotor and stator.

**Figure 6:**
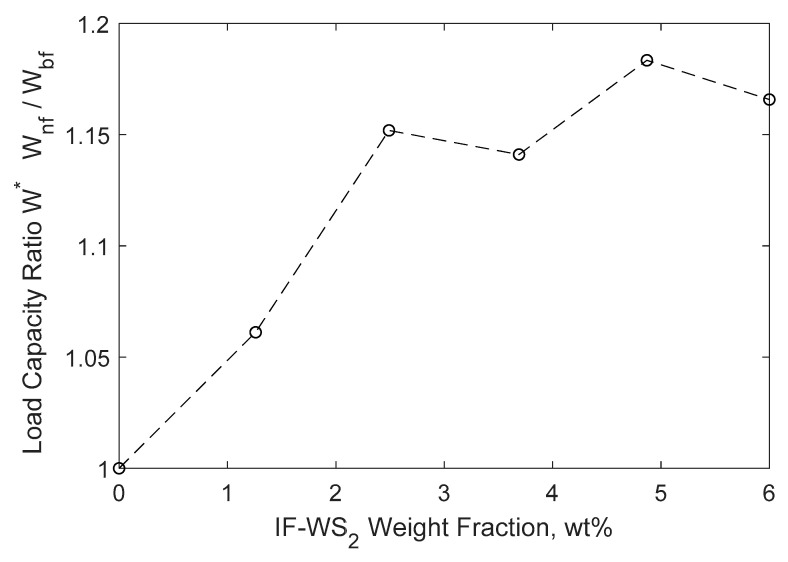
The relationship between the load capacity ratio of the journal bearings and weight fraction of the IF-WS_2_ nanofluid lubricant.

The integration of the pressure was used to calculate the load capacity of the bearing as per Equation (5). The results in Figure 6 show that generally increasing the wt% of nanofluid improves load capacity ratio, W *—the ratio of load capacity of the nanofluid ($w_{nf}$) to base fluid ($w_{bf}$). From the results, an 18% improvement was observed in Case 5, where 4.9 wt% of NP was used in the nanofluid lubricant. 

As previously mentioned, there are a few works that experimentally studied the effects of WS_2_ nanofluid lubricant on load carrying capacity of journal bearings [24,25], despite the fact that their experimental data were obtained using standard reciprocating ball/pin on disk tester. In order to assess the simulation findings presented in this work, the results were compared with published experimental data by Jian et al. [25] which are a closer fit to this work. They have shown that the maximum load that, beyond which wear is induced on the testing plate, could increase from ~510 N (for base oil) to ~597 N (for the oil containing 2.0 wt% WS_2_ nanosheets), showing an improvement of about 17% in load capacity. This result is comparable with our findings, which shows about an 18% increase, even though the test setup and type of WS_2_ NPs are different between our simulation and their published experimental results.

As it can be seen from the results in Figure 6, the load capacity ratio for 3.7 wt% and 6.0 wt% does not follow the typical increasing trend and declines slightly instead. It has been mentioned earlier that NP weight fractions for 6.0 wt% and higher may not be even applicable due to high particle aggregation. Even though the particle aggregation growth has not been modeled in this study, but the effect of particle collisions and particle-particle interactions are being considered in the DPM simulation of NPs. The higher weight fraction of NPs in nanofluid, the higher the likelihood of particle–particle collision of large aggregates. Such collisions have a negative effect on the flow momentum that result in either dropped maximum pressure (P_max_) or narrow the pressure profile observed in Figure 5. The main factors that alter the pressure profile are: (i) nanofluid relative viscosity and (ii) particle–particle collision. They have opposing influence on pressure profile shape and magnitude and act simultaneously on the model. This depends on their significance in each simulation case, and one of them could be the dominant factor towards reshaping the pressure profile that finally leads to the improvement or decline in the bearing load capacity. This could be the reason behind the declined load capacity ratio that was observed for few wt% in Figure 6. Further investigations, such as experimental studies in a lab setting, are needed to fully identify and evaluate this observation.

## 4. Conclusions

In this work, the CFD simulation approach was employed to model the effect of nanofluid lubricant on load carrying capacity of high-load industrial journal bearings. Inorganic fullerene-like WS_2_ nanoparticles were used as an additive to the base synthetic industrial oil with different weight fraction percentages. A 2D CFD model was developed and evaluated by comparing the simulation results with available theorical solutions. Nanoparticles were modeled using a DPM module. For the simulation purpose, nanofluid viscosity was calculated, taking into account the aggregation effect of NPs quantified using SEM imaging. The simulation was implemented for six different nanofluid scenarios (various WS_2_ weight fractions) to obtain the pressure profile of the hydrodynamic lubrication and to calculate the load capacity of the bearing. The results showed that the load carrying capacity of the journal bearing can be improved up to about 20% using a practical 5 wt% IF-WS_2_ NP additive. In addition, the SEM images show that the high aggregation tendency of WS_2_ NPs limits the application of nanofluid lubricant at weight fractions above 6.0 wt%. Further investigations could be done by employing a 3D simulation model to capture boundary and mixed lubrication regimes. In addition, extensive experimental studies and testing under various wt% are needed to fully identify the effect of WS_2_ nanofluid and NP aggregation on the load carrying capacity of rotating bearings. 

## Figures and Tables

**Table 1:** Parameters of the journal bearing used in the Computational fluid dynamics (CFD) simulation.

Journal Bearing Parameter	Value
Rotor radius	R = 19 mm
Rotor Length	L = 76 mm
Rotor angular speed	N = 200 rpm
Radial clearance	C = 0.038 mm
Base Lubricant viscosity (@100 °C)	µ = 0.02756 Pa s
Oil Density	ρ = 885 kg/m^3^

**Table 2:** Simulation cases of different weight fractions of the WS_2_ nanoparticles (NPs) investigated in this study.

Nanofluid Model	Weight Fraction (wt%)	Volume Fraction % (φ%)
**Case 1: No additive**	0%	0%
**Case 2**	1.26%	0.15%
**Case 3**	2.49%	0.30%
**Case 4**	3.69%	0.45%
**Case 5**	4.87%	0.60%
**Case 6**	6.02%	0.75%
